# Drive for muscularity and muscularity-oriented disordered eating in men: the role of set shifting difficulties and weak central coherence

**DOI:** 10.1186/2050-2974-1-S1-O49

**Published:** 2013-11-14

**Authors:** Scott Griffiths, Stuart Murray, Stephen Touyz

**Affiliations:** 1 University of Sydney, Australia; 2 Redleaf Practice, Australia

## 

Set shifting difficulties and weak central coherence are information-processing biases associated with thinness-oriented eating and body image pathology in women. However, little is known about the relationship between these processing biases and muscularity-oriented eating and body image pathology. We investigated whether set shifting and central coherence were uniquely related to the drive for muscularity and muscularity-oriented disordered eating in a sample of 91 male undergraduates. Participants completed the Wisconsin Card Sort Test, the Matching Familiar Figures Task, the Drive for Muscularity scale, and a modified Eating Disorders Examination - Questionnaire. Results indicated that set shifting difficulties and weak central coherence were both uniquely positively associated with the drive for muscularity, and that set shifting difficulties were uniquely positively associated with muscularity-oriented disordered eating. Results are discussed with regard to the male experience of body image and eating pathology, and in regard to muscle dysmorphia.

This abstract was presented in the **Body Image** stream of the 2013 ANZAED Conference.

